# Effect of body mass index and length of the disorder on hypoglycaemia in Anorexia Nervosa: continuous glucose monitoring in real-world settings

**DOI:** 10.1192/j.eurpsy.2026.12030

**Published:** 2026-07-31

**Authors:** A. Pulini, M. Septier, C. Westphalen, O. Viltart, P. Gorwood, P. Duriez

**Affiliations:** 1 INSERM U1266, Institute of Psychiatry and Neuroscience of Paris; 2 Clinique des Maladies Mentales et de l’Encéphale, GHU Paris Psychiatrie et Neurosciences, Hôpital Sainte Anne; 3 UFR médecine, Université Paris Cité, Paris; 4 CNRS UMR 9193, 3SCALab - Sciences Cognitives et Sciences Affectives; 5 PsySEF Faculty, Université de Lille, Villeneuve d’Ascq; 6 Clinique des Maladies Mentales et de l’Encéphale, GHU Paris Psychiatrie et Neurosciences Hôpital Sainte Anne, Paris, France

## Abstract

**Introduction:**

Anorexia nervosa (AN) often persists for years resulting in high morbidity and mortality rates. Hypoglycaemia, usually assessed from a single blood sample in the morning, is a critical severity indicator, continuous glucose monitoring (CGM) can therefore provide valuable data.

**Objectives:**

This study aims to provide new glycaemic characteristics and look for their drivers, such as metabolic (present Body Mass Index, BMI) and duration of illness, in a sample of patients tested in real-world settings.

**Methods:**

This cross-sectional study involved female outpatients with restricting subtype AN. Participants underwent CGM over 5 days in their usual environment. Data collected included age, BMI, illness duration, Eating Disorder Inventory-2 (EDI-2) scores. Glycaemic biomarkers such as hypoglycaemic area under the curve (AUC), mean and minimal glycaemia, and coefficient of variation were computed.

**Results:**

Three hundred female patients were recorded for an average of 4.8 days. No significant correlation was found between glycaemic biomarkers and BMI. Illness duration significantly correlated with mean and minimum glycaemia (r = 0.26 and 0.23, respectively, p < 0.001) and hypoglycaemia AUC (r = -0.25, p < 0.001). Prolonged illness duration was associated with lower hypoglycaemia AUC and higher mean and minimal glycaemia.

**Conclusions:**

In female patients with restricting subtype AN, illness duration rather than BMI may significantly impact glycaemic profiles. These results may suggest that glycaemic adaptations could be a factor that maintains undernutrition. AN should once more be conceptualized as a metabolic-psychiatric disorder.

**Disclosure of Interest:**

None Declared

